# Two centuries of vaccination: historical and conceptual approach and future perspectives

**DOI:** 10.3389/fpubh.2023.1326154

**Published:** 2024-01-09

**Authors:** David A. Montero, Roberto M. Vidal, Juliana Velasco, Leandro J. Carreño, Juan P. Torres, Manuel A. Benachi O., Yenifer-Yadira Tovar-Rosero, Angel A. Oñate, Miguel O'Ryan

**Affiliations:** ^1^Departamento de Microbiología, Facultad de Ciencias Biológicas, Universidad de Concepción, Concepción, Chile; ^2^Centro Integrativo de Biología y Química Aplicada, Universidad Bernardo O'Higgins, Santiago, Chile; ^3^Programa de Microbiología y Micología, Instituto de Ciencias Biomédicas, Facultad de Medicina, Universidad de Chile, Santiago, Chile; ^4^Instituto Milenio de Inmunología e Inmunoterapia, Facultad de Medicina, Universidad de Chile, Santiago, Chile; ^5^Unidad de Paciente Crítico, Clínica Hospital del Profesor, Santiago, Chile; ^6^Programa de Formación de Especialista en Medicina de Urgencia, Universidad Andrés Bello, Santiago, Chile; ^7^Programa de Inmunología, Instituto de Ciencias Biomédicas, Facultad de Medicina, Universidad de Chile, Santiago, Chile; ^8^Departamento de Pediatría y Cirugía Pediátrica, Facultad de Medicina, Universidad de Chile, Santiago, Chile; ^9^Área de Biotecnología, Tecnoacademia Neiva, Servicio Nacional de Aprendizaje, Regional Huila, Neiva, Colombia; ^10^Departamento de Biología, Facultad de Ciencias Naturales, Exactas y de la Educación, Universidad del Cauca, Popayán, Colombia

**Keywords:** vaccines, history of vaccines, vaccinology, types of vaccines, vaccine development, health literacy, vaccine hesitancy

## Abstract

Over the past two centuries, vaccines have been critical for the prevention of infectious diseases and are considered milestones in the medical and public health history. The World Health Organization estimates that vaccination currently prevents approximately 3.5–5 million deaths annually, attributed to diseases such as diphtheria, tetanus, pertussis, influenza, and measles. Vaccination has been instrumental in eradicating important pathogens, including the smallpox virus and wild poliovirus types 2 and 3. This narrative review offers a detailed journey through the history and advancements in vaccinology, tailored for healthcare workers. It traces pivotal milestones, beginning with the variolation practices in the early 17th century, the development of the first smallpox vaccine, and the continuous evolution and innovation in vaccine development up to the present day. We also briefly review immunological principles underlying vaccination, as well as the main vaccine types, with a special mention of the recently introduced mRNA vaccine technology. Additionally, we discuss the broad benefits of vaccines, including their role in reducing morbidity and mortality, and in fostering socioeconomic development in communities. Finally, we address the issue of vaccine hesitancy and discuss effective strategies to promote vaccine acceptance. Research, collaboration, and the widespread acceptance and use of vaccines are imperative for the continued success of vaccination programs in controlling and ultimately eradicating infectious diseases.

## 1 Introduction

Over the past century, a significant number of infectious diseases have been prevented, primarily due to advancements in science and technology. Among these breakthroughs, vaccines stand out as one of the most pivotal achievements in medicine and public health (Box 1). More than two centuries have passed since Benjamin Jesty and Edward Jenner laid the groundwork for vaccinology with their observations and experiments on smallpox and cowpox. Their pioneering efforts paved the way for the development of effective strategies for controlling and eradicating infectious diseases, many of which were considered invincible at the time.

Box 1 **What is a vaccine?**

A vaccine is defined as a biological product designed to stimulate the immune system to generate antigen-specific immunity against a pathogen, thereby preventing the disease it causes. Typically, vaccines are formulated from attenuated or inactivated versions of the pathogen, or derived components such as proteins and polysaccharides.

The addition of an adjuvant in many vaccine formulations serves to enhance the adaptive immune response.

Upon administering a vaccine, the immune system identifies some components of the pathogen (antigens) present in the vaccine producing a specific immune response.

Thus, the vaccine “trains” and prepares the immune system to respond effectively to the pathogen upon exposure; this phenomenon is known as immunological memory.

Therefore, when a vaccinated individual is later exposed to the same pathogen, their immune system will be prepared to generate an effective defense, preventing the development of the disease, or reducing its severity.

Each vaccine is meticulously designed and rigorously tested to ensure it elicits a specific immune response that is both safe and protective. This underscores the intricate balance and interaction between the vaccine composition and the dynamics of the immune system.

A century ago, infectious diseases were the primary cause of death worldwide. In 1900, the average life expectancy at birth in the United States was ~47 years, and children under five accounted for 30.4% of all deaths (1, 2). Survivors of these infections often suffered severe complications and disabilities such as paralytic poliomyelitis (3), osteomyelitis variolosa (4), and neurological and vision impairments, among others (5, 6). However, there was a significant decline in the mortality rate from infectious diseases throughout the 20th century, from 797 deaths per 100,000 in 1900 to 59 deaths per 100,000 in 1996 (7). By the late 1990s, chronic diseases like cardiovascular disorders, stroke, and cancer had become the leading causes of death (7). Currently, the average life expectancy at birth in the United States is ~78 years, marking an impressive 30-year increase (8). This trend is similarly observed in most middle- and high-income countries (9, 10).

The increase in life expectancy and the decline in mortality from infectious diseases can be attributed to various factors. Key among these is the reduction in disease transmission and host susceptibility, a consequence of improved housing, enhanced hygiene and sanitation, secure food and water supplies, and the widespread use of safe, effective, and affordable vaccines. Additionally, significant advances in medical treatments, including antimicrobial and antiviral agents, have contributed substantially (11).

Collectively, these advances in public health have markedly contributed to the eradication of important pathogens, such as smallpox virus and wild poliovirus types 2 and 3 (with wild polio type 1 close to eradication) (12–14). Several vaccine-preventable diseases, including diphtheria, measles, mumps, rubella, and pertussis, are now largely under control. Nonetheless, the path toward a world free of these infectious diseases is complex and faces significant challenges, making it essential to maintain adequate vaccination coverage to avoid resurgences (15–17).

Numerous infectious diseases continue to afflict humanity, and while significant progress has been made in some areas, notable gaps remain in our vaccine arsenal. One of the most prominent examples is HIV/AIDS, a global pandemic that has persisted for decades. Despite extensive research, concerted efforts, and numerous clinical trials, an effective HIV vaccine remains elusive (18). This scenario underscores the complexity and challenges of vaccine development against certain pathogens, even with advances in modern science. These challenges highlight the urgent need for continued support for research and innovation in vaccinology.

It is worth noting that some of the leading causes of child mortality, such as malaria and respiratory syncytial virus (RSV), are soon to be tackled with prevention strategies that will include new vaccines (19–21). Additionally, the persistent threat of emerging and reemerging diseases, as demonstrated by the recent COVID-19 pandemic, further accentuates the need for advancements in vaccinology. These advancements, supported by cutting-edge genetic engineering, molecular biology, and structural biology, have expedited the development of several innovative vaccines against SARS-CoV-2.

However, the challenges we face are not purely biological. During the COVID-19 pandemic, an “infodemic” occurred, characterized by the spread of false, misleading, or biased information related to vaccines (22). In this context, it becomes imperative to promote accurate and evidence-based information to achieve broad acceptance and understanding of vaccines within communities.

This narrative review aims to trace the path of historical milestones in the development and progress of vaccines, recognizing pioneers with global impact in this field. We will briefly explain the principles and mechanisms of action of the main types of vaccines, highlighting their characteristics, advantages, and limitations. Additionally, we analyze the impact of vaccines, emphasizing their contribution to reducing morbidity and mortality, as well as their economic and social benefits. Finally, we address the issue of vaccine hesitancy and underscore the importance of effective communication to promote vaccination acceptance.

Aimed primarily at non-expert audiences in the healthcare field, this review seeks to provide useful information to improve health literacy and better address the growing threat of vaccine misinformation. Ultimately, the acceptance and widespread use of vaccines are *sine qua non* conditions for further progress in controlling and eradicating infectious diseases.

## 2 Methodology

For this narrative review, a comprehensive literature search was carried out in the PubMed, Science Direct, and Google Scholar databases. The search strategy was formulated using a combination of keywords: “vaccine development history,” “vaccine types,” “immune response to vaccines,” “vaccine public health impact,” and “vaccine hesitancy.” This set of keywords was selected to ensure the inclusion of a broad range of relevant articles covering various aspects of vaccinology. The abstracts of the articles were then reviewed to evaluate their relevance and eligibility based on the inclusion criteria. Selection criteria were defined to include articles that described historical milestones in vaccine development, addressed the immunological basis of vaccination, or discussed the origin, causes and mitigation strategies of vaccine hesitancy. Articles that met these criteria were reviewed in their entirety. In addition to database searches, the reference lists of the selected articles were hand searched to identify further relevant studies that may not have been included in the database searches. This literature search and article selection approach was designed to ensure that the review was comprehensive and unbiased, providing a well-rounded perspective on the history, development, and impact of vaccines on public health.

## 3 History of vaccines and vaccination

Most stories in microbiology usually begin with the first observation of microorganisms. Microorganisms were absent from human knowledge until 1674, when the Dutch merchant Antonie van Leeuwenhoek, a self-taught scientist, and naturalist, discovered the microscopic world (23, 24).

Leeuwenhoek, employing refined lenses of his own manufacture, meticulously documented the existence of “animalcules”, now known as bacteria and protozoa. His detailed observations, written and drawn in numerous letters addressed (almost always) to the Royal Society of London, provided the first images of cells and organisms that cannot be seen with the naked eye (23). These findings were foundational, paving the way for the emergence of scientific disciplines like cell biology and microbiology, which have their roots in understanding the microscopic world.

As we delve into the following sections, the fundamental role of the discovery of microorganisms in the field of vaccinology will become increasingly evident. However, to fully understand this impact, it is necessary to take a journey to an era before the invention of vaccines.

This historic analysis reveals a chronicle marked by perseverance, innovation, defeats, and triumphs, which collectively summarize the evolution of vaccines. This history not only deserves celebration but also serves as an axis that connects our past understandings, current knowledge, and projections in the fields of immunization and disease prevention.

### 3.1 Variolation, the ancient method of immunization

As we look through the annals of medicine, we encounter a period before the development of vaccines, a time when rudimentary methods by today's standards were used to fight infectious diseases. One such method was variolation, the practice of inoculating healthy individuals, either through the nose or a scratch in the skin, with material obtained from smallpox pustules to confer immunity (25, 26).

Smallpox, caused by the Variola virus, was a highly contagious disease, transmitted primarily through direct contact and respiratory droplets. The disease presented in two clinical forms. Variola major, the more common and severe form, was characterized by an extensive rash and high fever, and an overall mortality rate close to 30%. Variola minor was less prevalent and exhibited a milder manifestation, with mortality rates of 1% or less (27).

Variolization was practiced in Asia, particularly in China and India, as early as the 17th century AD, although it probably originated centuries earlier. Lu, a renowned Chinese physician, provided the first detailed description of variolation in a book published in 1695 (28). He described three main methods: the first involved inserting a piece of cotton soaked in pus from fresh pustules into the nostrils; the second consisted of inhalation of dried and powdered scabs; the third involved exposing a healthy individual to clothing worn by an infected individual. Each method induced a mild form of smallpox and subsequent immunity, with variolation being considered more effective and safer compared to natural infection exposure. The Chinese also distinguished between variola major and minor, extracting smallpox material from people affected by the latter. However, despite its relative efficacy, variolation was not without significant risks, including the possibility of suffering severe smallpox, and even death (26, 29).

In India, the variolation method was different; it involved inoculating individuals with smallpox material through a scratch in the skin (cutaneous inoculation). This method was recognized as safer than the Chinese practices and spread to the Middle East through merchant caravans (30–33).

In the 18th century, variolation found its way to Europe, mainly due to the efforts of Lady Mary Wortley Montague, the wife of the British ambassador to the Ottoman Empire. During her stay in Constantinople, Lady Montague learned about variolation. Having herself suffered from smallpox, she became a strong advocate for this preventive method. In 1721, after returning to London, she decided to variolate her 3-year-old daughter in the presence of the English court physicians. The successful protection of her daughter against smallpox, coupled with her strong advocacy for variolation, stimulated the adoption of this method throughout Europe (32, 34).

In North America, the promotion of variolation was notably led by Reverend Cotton Mather and Dr. Zabdiel Boylston, who fervently advocated for its use (35). Their advocacy was particularly crucial during a smallpox epidemic in Boston in 1721, which claimed hundreds of lives. Data from the United States National Library of Medicine indicates that 0.5–3% of those variolated died, compared to 9.5–30% dying from smallpox after natural exposure (36). Despite presenting comparative analyses of mortality rates pointing to its efficacy, proponents of variolation faced considerable opposition.

Benjamin Franklin, who was also personally affected by smallpox, joined the defense of variolation after losing his son to the disease in 1736. He deeply regretted not having variolated his son and conveyed his experience to other parents, urging them to choose variolation as the safest way to protect their children (37).

Despite its associated risks, variolation was an important step toward comprehending and developing techniques to prevent smallpox. Adopting and promoting this method through the efforts of prominent figures like Lady Montague, Reverend Mather, and Dr. Boylston, marked a significant advance in the history of public health. Although safer and more effective immunization strategies eventually replaced variolation, its historical significance is indelible. It represents the persistent search for strategies to fight infectious diseases.

### 3.2 Benjamin Jesty, Edward Jenner, and the foundation of vaccinology

In an era when smallpox ravaged populations, there was a desperate search for preventive methods more reliable and safer than variolation. In this historical context, vaccinology has its roots not only in the well-documented work of the English physician Edward Jenner but also in the lesser-known but significant contributions of the farmer Benjamin Jesty.

Jesty made the critical observation, as Jenner would years later, that milkmaids who had contracted cowpox (a disease similar but milder to human smallpox) did not contract smallpox, even after close contact with infected individuals. In 1774, during a smallpox outbreak in England, Jesty adeptly applied this observation and inoculated his wife and two sons with material from a cowpox pustule using a stocking needle. Jesty did not inoculate himself because he had previously contracted cowpox and was confident that he was already protected (38). This event is considered the first recorded vaccination. The successful result of this method was evidenced by the fact that his family never suffered from smallpox, even when they were subsequently exposed to the disease. Moreover, Jesty extended his efforts to vaccinate other individuals in his community (39, 40).

While Jesty's efforts were pioneering, Jenner's systematic experiments and published works earned him a unique place in history, as the “father of vaccinology”. As mentioned above, Jenner also noted apparent immunity to smallpox among individuals who had contracted cowpox. Prompted by this observation, Jenner performed a series of experiments involving the inoculation of material from cowpox pustules. In 1796, he inoculated James Phipps, an 8-year-old boy, with material from a fresh cowpox lesion obtained from a milkmaid named Sarah Nelms. Subsequently, when Jenner exposed the boy to material from a human smallpox lesion, Phipps did not become ill, demonstrating the protective capacity of this method (41, 42). Jenner compiled the findings of this experiment, along with sixteen additional case histories, into his publication “An inquiry into the causes and effects of the *variolae vaccinae*” (43). The success of these experiments demonstrated that cowpox minimally affected humans while generating protection against smallpox.

However, at the time, Jenner was unable to elucidate why his method provided protection, owing to an incomplete understanding of the causal relationship between microorganisms and diseases. As knowledge in microbiology and immunology advanced, later scientists adapted and expanded his fundamental work (34, 44, 45). Furthermore, the insights of Jenner into the essential role of animals in vaccinology were truly ahead of his time, foretelling the future use of cows, guinea pigs, rabbits, and even chicken eggs in vaccine development (46). However, the use of cows in Jenner's method made many people wary and sometimes hostile to the idea of inoculating foreign animal products into their own bodies. Initially, Jenner encountered satirical ridicule in the popular press and opposition from eminent physicians. Yet, as word of his breakthrough spread, his work gradually became accepted, acknowledged, and celebrated (46, 47).

Jenner's work based on scientific methods of observation and experimentation led to the formulation of the vaccine concept. The terms “vaccine” and “vaccination” originate from “*variolae vaccinae*”, a phrase coined by Jenner to literally refer to smallpox of the cow. In 1881, Louis Pasteur, known as the “father of microbiology,” in recognition of Jenner's legacy, proposed extending these terms to the new protective immunizations that were being developed at that time. Thus, the terms vaccine and vaccination transcended their origin and began to be applied to all biological products and methods used to confer immunity against infectious diseases (41, 48).

Importantly, the discoveries of Jenner revolutionized prevention of infectious diseases, influencing the development of all subsequent vaccines (29, 48). Therefore, while Jesty is recognized as the first vaccinator, it was Jenner who laid the foundations for the establishment of vaccinology as a scientific discipline. Table 1 presents a select list of vaccines developed after Jenner's seminal discovery.

**Table 1:** Outstanding examples of vaccines developed^*^.

**Pathogen**	**Disease**	**Year**	**Developer(s)**	**Vaccine type**	**References**
Variola virus	Smallpox	1796	Edward Jenner	Vaccine based on bovine smallpox virus	(43)
Rabies virus	Rabies	1885	Louis Pasteur and Émile Roux	Attenuated vaccine	(49)
*Salmonella enterica* Serovar Typhi	Typhoid fever	1896	Richard Pfeiffer and Almroth Wright	Inactivated vaccine	(50)
*Vibrio cholerae*	Cholera	1896	Wilhelm Kolle	Inactivated vaccine	(51)
*Yersinia pestis*	Bubonic plague	1897	Waldemar Haffkine	Inactivated vaccine	(52)
*Mycobacterium tuberculosis*	Tuberculosis	1921	Albert Calmette and Camille Guérin	Attenuated vaccine based on *Mycobacterium bovis*	(53)
*Corynebacterium diphtheria*	Diphtheria	1923	Gaston Ramon	Toxoid vaccine that protects against the toxin	(54)
*Clostridium tetani*	Tetanus	1925	Gaston Ramon	Toxoid vaccine that protects against the toxin	(55)
*Bordetella pertussis*	Pertussis	1930s	Pearl Kendrick and Grace Eldering	Whole-cell inactivated vaccine	(56)
Yellow fever virus	Yellow Fever	1937	Max Theiler	Attenuated vaccine (17D strain)	(57)
Polio virus	Poliomyelitis	1955	Jonas Salk	Inactivated vaccine that protects against all 3 serotypes	(58)
Polio virus	Poliomyelitis	1960	Albert Sabin	Oral attenuated vaccine that protects against all 3 serotypes	(59)
Measles virus	Measles	1954–1960	John F. Enders and Samuel L. Katz	Attenuated vaccine; part of the MMR vaccine	(60)
Mumps virus	Mumps	1967	Maurice Hilleman	Attenuated vaccine; part of the MMR vaccine	(61)
Rubella virus	Rubella	1969	Stanley Plotkin	Attenuated vaccine (RA 27/3 strain); part of the MMR vaccine	(62)
Varicella-Zoster virus	Varicella	1974	Michiaki Takahashi	Attenuated vaccine (Oka strain)	(63)
*Neisseria meningitidis* serogroups A, C, W, and Y	Meningitis	1981		Polysaccharide vaccine	(64, 65)
Hepatitis B virus	Hepatitis B	1982	Baruch Blumberg and Irving Millman	Subunit vaccine based on viral surface protein	(66)
*Streptococcus pneumoniae*	Pneumonia	1983	Robert Austrian et al.	Polysaccharide vaccine against 23 serotypes	(67)
*Haemophilus influenzae* type b	Pneumonia, meningitis, and other illnesses	1985	David H. Smith, Porter Anderson, et al.	Polysaccharide vaccine	(68)
*Haemophilus influenzae* type b	Pneumonia, meningitis, and other illnesses	1987		Conjugate polysaccharide vaccine	(69)
*Vibrio cholerae*	Cholera	1991	Jan Holmgren et al.	Vaccine containing killed whole cell of *V. cholerae* O1 and cholera toxin B subunit	(70)
*Bordetella pertussis*	Pertussis	1992	Rino Rappuoli et al.	Acellular vaccine	(71)
Hepatitis A virus	Hepatitis A	1990s	Various developers	Inactivated vaccines	(72)
*Neisseria meningitidis* serogroup C	Meningitis	1999		Conjugate polysaccharide vaccine	(73)
*Streptococcus pneumoniae*	Pneumonia	2000		Conjugate polysaccharide vaccine against seven serotypes	(74)
*Neisseria meningitidis* serogroups A, C, W, and Y	Meningitis	2005		Conjugate polysaccharide vaccine	(65)
Rotavirus	Gastroenteritis	2006	Various developers	Attenuated vaccine against rotavirus and reassortant vaccine	(75)
Human Papillomavirus (HPV)	Human papillomavirus-associated cancers	2006	Ian Frazer and Jian Zhou	Subunit vaccine based on viral proteins; protects against cervical cancer and other HPV-associated cancers	(76)
*Neisseria meningitidis* serogroup B	Meningitis	2013		Subunit vaccine plus outer membrane vesicles.	(77)
SARS-CoV-2	COVID-19	2020–2021	Various developers	Various technologies: inactivated vaccines, mRNA vaccines, and non-replicating viral vector vaccines.	(78–85)
Respiratory syncytial virus (RSV)	Cold-like symptoms, pneumonia.	2023		Subunit vaccine based on the prefusion F protein.	(86, 87)

^*^For a historical context, the first vaccines to be licensed or those that marked a milestone in the management of a specific disease are highlighted. The approximate year of development or licensure and the main developers are indicated. The optimization of many of these vaccine formulations has led to their replacement by others that have proven to be safer and more effective. The names of the main developers of the vaccine are indicated. In some cases, the vaccines were developed by pharmaceutical companies and therefore their names are omitted. For more information refer to the text.

In 1980, the World Health Organization (WHO) declared the eradication of smallpox. This is one of the most outstanding achievements of all time in public health and science, demonstrating the power of vaccination in the fight against infectious diseases. In addition, it underscored the relevance of cooperation between scientists, institutions, and governments in providing extraordinary outcomes for the benefit of humankind (34, 88).

### 3.3 The contribution and impact of Louis Pasteur

Between the 1850s and 1860s, the French chemist Louis Pasteur conducted a series of groundbreaking experiments that substantiated the Germ Theory. He conclusively demonstrated that food spoilage was due to the presence and contamination of organisms that cannot be seen with the naked eye (microorganisms), thereby discrediting the idea of spontaneous generation (89).

His investigations also led to the development of experimental techniques to mitigate the deleterious effects of microorganisms in foods and beverages. From 1860 to 1864, he worked on the pasteurization method, which involves heating liquids to a specific temperature for a defined period to eliminate or significantly reduce the presence of harmful microorganisms (89–91). Initially applied to wine and beer, this method not only extended their shelf life but also ensured their safety for consumption. The adaptation of the pasteurization method to milk significantly reduced the transmission of milk-borne diseases (91).

In 1864, Pasteur proposed the “Germ Theory of Disease”, postulating that infectious diseases were caused by microorganisms (92). This theory laid the foundations for understanding how infectious diseases spread among people through the transmission of pathogenic microorganisms. However, this approach was subject to intense debate during the following decades, and various versions of the germ theory of disease continued to circulate (93).

It was not until the late 19th century, with Robert Koch, that consensus was reached for this theory. Koch identified the causative agent of anthrax and later tuberculosis (see below). Based on his findings, he established the criteria (Koch's postulates) as a requirement to establish a causal relationship between a microorganism and the development of a specific disease (94).

In 1877, Pasteur began studies on avian cholera (also called fowl cholera), identifying *Pasteurella multocida* as the bacterium that causes this disease. In 1879, he accidentally discovered that cultures of this bacterium experienced a decrease in virulence over time (95). In a serendipitous twist of events, Pasteur, before leaving for vacation, instructed an assistant to inject some chickens with fresh cultures of *P. multocida*, but the assistant forgot to do so before leaving for vacation. Upon return, the assistant inoculated the chickens with the cultures that had been left in the laboratory for a month in glass tubes sealed only with a cotton plug. Contrary to expectations, the chickens developed mild symptoms and fully recovered. Intrigued, Pasteur injected the recovered chickens with an inoculum of fresh culture of *P. multocida* awaiting for the development of the disease. Observing that the birds remained healthy, he deduced that exposure to oxygen caused the loss of virulence. To validate this hypothesis, a series of controlled experiments were conducted. As a result, it was observed that *P. multocida* cultures that were tightly sealed and isolated from air maintained their virulence. In contrast, those exposed to air for varying durations exhibited a consistent and predictable decline in their virulent nature. Pasteur named this reduction in virulence “attenuation”, a term that remains today (95). Pasteur also observed that some infected albeit healthy chickens excreted virulent *P. multocida*, indicating the existence of healthy carriers, a key concept for explaining the spread of germs during epidemics (90).

In 1880, Pasteur in France and George Miller Sternberg in the United States simultaneously isolated *Streptococcus pneumoniae*. This bacterium is responsible for various human diseases, including pneumonia, bacteremia, meningitis, empyema, and endocarditis (96).

The following year, Pasteur, with his colleagues Charles Chamberland and Emile Roux, developed an attenuated vaccine against *Bacillus anthracis*, a serious threat to the sheep industry. In contrast to the *P. multocida* cultures, *B. anthracis* cultures transformed into highly virulent spores when exposed to air. However, *B. anthracis* strains grown at a temperature of 42–43°C did not form spores. Although these non-sporulated cultures remained live at these temperatures for a month, a pronounced reduction in virulence was observed following administration to animals (95, 97). Another key finding by Pasteur and colleagues in their research on chicken cholera and anthrax was that repeatedly transferring (serial passage) a microorganism through the same or a different animal species could change its ability to cause disease, either increasing or reducing its virulence (89, 98).

During the 1880s, Pasteur achieved another breakthrough in vaccinology by developing the rabies vaccine. Rabies is a zoonotic disease that primarily affects mammals, including humans, and is transmitted mainly through the bite of infected animals. The rabies virus attacks the central nervous system, causing encephalitis with a very high lethality rate (99).

At the time, the Latin-derived term “virus”, which means “poison”, was employed to denote any agent that caused infectious disease. The ability to visualize viruses did not emerge until the invention of the electron microscope 50 years later in the 1930s (100). Notwithstanding the lack of clarity on the distinction between bacteria, fungi, and viruses, Pasteur made substantial advancements through his nuanced understanding of disease-causing agents and immunity. Notably, fine filtration techniques devised by Pasteur allowed for the differentiation between microbes. Those of larger size that could be cultivated outside the body (*in vitro*) and observed to form colonies visible to the naked eye were classified as bacteria. By contrast, pathogens that passed through these smaller filters and were not cultivable outside of living cells became known as viruses. This provided a working definition for viruses, valid until the mid-20th century when the electron microscope facilitated their visualization (89).

For the rabies vaccine, Pasteur recognized that the virus could not be cultivated *in vitro* as it was an actual virus and not a bacterium; thus, the method of atmospheric attenuation could not be used. Instead, he relied on his understanding of the serial passage of microorganisms from one animal to another. In collaboration with his students, Pasteur developed the rabies vaccine by desiccating nervous tissue from rabbits infected with rabies. The virulence of the pathogen decreased progressively during 14 days of desiccation and through successive passages. This led Pasteur to discover that this attenuated virus could protect animals (rabbits or dogs) against a challenge with the wild-type virus without inducing severe disease (89).

In 1885, a 9-year-old boy named Joseph Meister was bitten by a rabid dog and brought to Pasteur's laboratory. Even though the vaccine had not been tested in humans, Pasteur decided to administer it to the child due to the gravity of the situation (29, 49). It is important to note that the rabies virus has a prolonged and variable incubation period that ranges from 4 to 12 weeks or more. Thus, in the case of a bite from an infected animal, the virus does not immediately cause the disease (101). This time between virus entry and symptom onset (today known as the incubation period) provides a window for vaccine administration and the generation of protection. Following this rationale, Meister received a vaccination series during the incubation period. The child did not develop the disease and fully recovered. This marked the birth of the first successful vaccine against rabies and the beginning of a new era in preventing infectious diseases (29). Following this pioneer rabies vaccine, carbolic acid-inactivated nerve tissue-derived vaccines were introduced, followed by phenol-inactivated versions in 1915. These vaccines were used until the mid-1950s when tissue culture-derived inactivated rabies vaccines were first developed, which remain in use today (89, 99, 102).

It should be noted that Pasteur conducted his entire vaccine research without an understanding of the biological processes involved in the protection of vaccinated animals and individuals. However, his work represents the development of the first laboratory-created vaccines, leading to the “isolate, inactivate, and inject” principle that underpinned vaccine development for the next century (95, 103–105).

The legacy of Pasteur goes beyond his revolutionary scientific discoveries, toward an institutional influence. In 1888, the Pasteur Institute was founded, a center dedicated to rabies, as well as research and training in infectious diseases (106). Named after Pasteur, the institute continues its mission to prevent and treat diseases through research, education, and public health intervention.

The last decade of the 19th century marked the beginning of an era in which vaccine development was supported by more solid scientific principles. This progress was led by eminent scientists from Great Britain, Germany, the United States, and Pasteur's laboratory in France. Key achievements of this decade included techniques for inactivating whole bacteria and their use as vaccines (killed vaccines; see below), the discovery of bacterial toxins, and of immune serum containing antibodies capable of neutralizing toxins, denominated antitoxins (103).

During this period, inactivated whole-cell vaccines against diseases such as typhus, cholera, and plague were developed and successfully tested (50–52, 107, 108). Emil von Behring, Shibasaburo Kitasato, Émile Roux, Alexandre Yersin, Almwroth Wright, and Paul Ehrlich are a few of the leading researchers in the field of serum antibodies. Ehrlich, in particular, expanded understanding of antibodies as complementary entities to antigens. Additionally, Roux and Yersin demonstrated that diphtheria bacilli produced an exotoxin, and von Behring and Kitasato verified that antitoxin antibodies could be induced in animal sera exposed to sublethal doses of toxin (103, 109–111).

### 3.4 The dawn of the 20th century, the discovery of toxoids, and the development of a vaccine for tuberculosis

Before the 20th century, diseases such as diphtheria, tetanus, pertussis, and tuberculosis were major causes of morbidity and mortality, and effective treatments or adequate preventative measures were unavailable.

Diphtheria, a potentially fatal disease, is caused by the bacterium *Corynebacterium diphtheriae*. This pathogen primarily affects the upper respiratory tract and produces a toxin (diphtheria toxin) that disrupts cellular function causing exudative pharyngitis followed by systemic involvement (112). Tetanus is a severe nervous system infection caused by the bacterium *Clostridium tetani*, commonly found in the soil. This bacterium produces a neurotoxin (tetanus toxin) which can cause muscle contractions, including violent spasms, leading to death in severe cases (113).

In 1923, Alexander Glenny and Barbara Hopkins made a significant scientific breakthrough by demonstrating that diphtheria toxin could be inactivated into a toxoid using formalin. Although the toxicity of the toxin was significantly reduced, it was not abolished, and in order to be well-tolerated, it required administration with an antitoxin serum (109, 114). Later, Gaston Ramon was able to produce a stable and non-toxic diphtheria toxoid through the action of formalin and subsequent incubation at 37°C for several weeks. Immunization with this toxoid generated protective antibodies against the diphtheria toxin, laying the foundation for an effective vaccine. This same procedure was used to prepare the tetanus toxoid and several other toxoids (54, 55, 109, 115).

Pertussis, also known as “whooping cough,” is caused by the bacterium *Bordetella pertussis*. This infection affects people of all ages, potentially causing severe disease in infants and death. In the early efforts against pertussis, the work of Thorvald Madsen in the 1920s led to a formalin-inactivated whole-cell vaccine that provided a degree of protection, but it was the work of Pearl Kendrick and Grace Eldering in the 1930s which finally provided an effective vaccine against whooping cough (56, 116). In 1948, vaccines against diphtheria, tetanus, and whooping cough were combined into the DTP vaccine, leading to a significant decrease in associated illnesses and deaths (117, 118). Due to pertussis toxin content, the vaccine was associated with considerable side effects such as fever, inflammation at the injection site, and in rare cases, severe neurological disorders, including encephalopathy (17, 119). Concerns about the safety of this vaccine led in the following decades to the development of less reactogenic formulations through endotoxin removal in acellular formulations as reviewed further down.

One of the “global killers” has been and continues to be tuberculosis (TB), named by Johann Schonlein in 1834, and referred throughout history as: “phthisis” in ancient Greece, “tabes” in ancient Rome, and “schachepheth” in ancient Hebrew. In the 18th century, it was denominated “the white plague” due to the characteristic pallor of affected individuals. Although Schonlein had already named it tuberculosis, in the 19th century, it was also called “consumption”. During this period, TB acquired the grim nickname of “Captain of all these men of death” (120, 121).

TB primarily affects the lungs but can also affect other organs. It is transmitted airborne when a person with active TB coughs, sneezes or speaks (122). In 1882, Robert Koch identified *Mycobacterium tuberculosis* as the bacterium responsible for TB (123). TB was one of the leading causes of death at that time, affecting one out of seven individuals in the United States and Europe (120).

Years later, in 1921, Albert Calmette and Camille Guérin developed the Bacille Calmette-Guérin (BCG) vaccine based on an attenuated strain of *Mycobacterium bovis*, a bacterium closely related to *M. tuberculosis* (53). This vaccine was developed in a remarkable effort through 230 serial passages of *M. bovis* in medium containing bile, over a period of 13 years (124, 125). This rigorous procedure allowed for the selection of avirulent strains lacking the ability to cause disease. Later work by Calmette and Guérin demonstrated that their vaccine protected animals and infants against *M. tuberculosis* (103, 125).

Although the BCG vaccine offers critical protection against severe forms of TB in children, such as military tuberculosis and tuberculous meningitis, its efficacy against pulmonary TB in adults has been inconsistent (126). The genetic variability between different BCG vaccine strains and the variable protection observed in different populations and geographic regions further underscore the complexities of tuberculosis immunity (125). Moreover, there is a pressing call within the scientific community for the development of new TB vaccines. However, this endeavor has been hampered by a myriad of challenges, including our limited understanding of the correlates of protective immunity against TB (127), the pathogen's sophisticated immune evasion strategies, and the multifaceted nature of the disease itself (128).

Despite the availability of BCG vaccine and several antibiotics, the control of TB is currently hindered by the emergence of multidrug-resistant strains of *M. tuberculosis*, especially in vulnerable populations such as immunocompromised individuals (129). This persistent challenge underscores the urgent need for novel TB vaccine candidates and advanced therapeutic approaches. Global initiatives focusing on prevention, early detection, and effective treatment are essential to reduce the burden of TB and advancing toward the potentially achievable, albeit difficult goal of eradication (130).

During the 1930s, the serial passage technique, either *in vitro* or in unusual hosts, was continually employed to attenuate various pathogens. For example, Max Theiler and Hugh Smith attenuated the yellow fever virus by serial passage in mice and chicken embryo tissues, respectively (57, 131, 132).

### 3.5 Second half of the 20th century and the eradication of poliomyelitis

In the second half of the 20th century, vaccinology made considerable achievements, mainly due to the introduction of novel methodologies for vaccine development. Among these, tissue culture methods allowed the controlled growth of bacteria and replication of viruses in the laboratory. This advancement significantly accelerated the large-scale production of vaccines (133).

These advances were complemented by improvements in storage and distribution systems, highlighted by applying preservatives and incorporating the cold chain. This ensured the quality and viability of vaccines during their storage and transport. Importantly, these advances facilitated the distribution of vaccines, providing access to an ever-increasing number of individuals worldwide (134).

A hallmark achievement during this period of rapid scientific evolution was the successful control and near-eradication of poliomyelitis. This viral disease, known to cause paralysis and permanent disability, affected hundreds of thousands of individuals annually at the time. Two significant contributors to this effort were Jonas Salk and Albert Sabin. In 1955, Salk developed the first inactivated polio vaccine (IPV), formulated with chemically inactivated viral particles encompassing all three poliovirus types (58, 135). However, IPV had inherent limitations, such as the need for administration via injection and booster doses owing to its reduced potency (136). Moreover, IPV faced some initial setbacks, including contamination of two production batches with viable viral particles, which led to serious health problems among those vaccinated and product recalls, and raised significant public doubts regarding its use. The production of IPV was resumed after stringent improvements in quality control measures and supervision (35).

A few years later, in 1961, Sabin developed an oral polio vaccine (OPV) based on attenuated viruses (59). This vaccine exhibited advantages over IPV in terms of ease of administration, cost-effectiveness, and provision of long-lasting immunity limiting the need for booster doses. Nevertheless, OPV was not without risks. On rare occasions, vaccination with the live attenuated virus could result in paralytic poliomyelitis—a condition termed vaccine-associated paralytic poliomyelitis (VAPP) or mutate to a more virulent strain causing small outbreaks of vaccine-derived poliovirus (VDPV). Despite these potential risks, the benefits of OPV resulted in its widespread adoption in Western regions, and it was instrumental in extensive vaccination campaigns that significantly decreased the global incidence of polio (12, 13).

By the end of the 1990s, the challenge was to balance the benefits and risks associated with OPV and IPV in a global plan for poliovirus eradication requiring the vaccination of the world population. As polio cases markedly declined, the relatively minor yet substantial risk of VAPP came into sharp focus, prompting recommendations for IPV usage in polio-free nations. In contrast, OPV continued to be used for routine immunization in regions where the disease remained more prevalent (137, 138). This transition illuminates a broader trend in the evolution of vaccinology: recognizing and addressing the inherent limitations and risks of vaccines to maximize their potential benefits.

In 2016, a global coordinated shift occurred from trivalent OPV (tOPV), containing Sabin strain types 1, 2, and 3, to bivalent OPV (bOPV), containing Sabin strain types 1 and 3. Remarkably, clinical cases of wild poliovirus have decreased by over 99% since 1988, with an estimated 350,000 cases in more than 125 endemic countries compared to only 6 cases reported in 2021 (12, 13, 138). Today, wild poliovirus type 1 is endemic only in Afghanistan and Pakistan, but there has been a rise in circulating vaccine-derived poliovirus type 2 outbreaks since 2017. In response to these outbreaks, in 2020, the WHO granted Emergency Use Listing for the novel oral poliovirus type 2 (nOPV2; genetically stabilized) to be used in a limited number of countries. The Polio Eradication Strategy for 2022–2026 outlines the wider use of nOPV2 to progress toward total eradication (12). The success of polio vaccines exemplifies the triumphs and challenges of modern vaccinology, reflecting the continuing importance of technological, logistical, and ethical considerations in the drive toward global health improvement. However, one of the main challenges will be to ensure optimal coverage of these vaccines, especially after the COVID-19 pandemic, in which coverage has decreased in many regions of the planet (139).

During the 1960s, important vaccines against prevalent viral diseases such as measles, rubella, and mumps were developed. Measles, a highly contagious infection, can be fatal by causing pneumonia and neurological complications (140). Although mumps is generally less lethal, it can cause severe complications, such as aseptic meningitis and encephalitis (141). On the other hand, rubella, while often mild in children, can have devastating effects on pregnant women and neonates (142).

The first approaches to developing vaccines against these pathogens focused on developing formalin-inactivated viruses. However, these vaccines failed to provide full and long-lasting immunity, so efforts turned to the development of attenuated vaccines (35). These vaccines were developed by weakening the viruses through their passage in embryonated eggs or cell cultures, making the attenuated viruses safe, and less reactogenic while retaining immunogenic capacity (62, 142, 143).

The first attenuated measles vaccine was developed by John Enders between 1954 and 1960 and later licensed in 1963 (60, 144, 145). At the same time, Maurice Hilleman and colleagues developed an attenuated mumps vaccine, approved in 1967 (61). Regarding rubella, Paul Parkman, and Harry Meyer Jr. developed the first attenuated vaccine in 1965, known as HPV-77 (143, 146). However, Hilleman developed a more effective vaccine, the RA 27/3 (62), which by the late 1970s became the only rubella vaccine used worldwide, except in Japan (147). Live attenuated rubella vaccine strains Takahashi, Matsuura, and TO-336 were licensed in Japan in 1969-1970 and continue to be used today (148, 149).

The 1970s ushered in the era of combination vaccines, particularly the combination of live vaccines into a single formulation offering protection against measles, mumps, and rubella (MMR vaccine) (150). MMR vaccine simplified immunization schedules and reduced the number of inoculations. Importantly, it exhibited substantial efficacy, resulting in a marked decline in the global incidence of these diseases (151). Before widespread vaccination against measles in 1980, this disease caused ~2.6 million annual deaths worldwide (152). After mass vaccination, measles deaths have drastically reduced, to ~140,000 deaths in 2018 (153).

In the same decade, Michiaki Takahashi developed the vaccine against the varicella-zoster virus by cultivating it serially in human embryonic lung cells and then in guinea pig embryo cells (63). However, this vaccine was not licensed until 1987 in Japan and Korea and not until 1995 in the United States and other countries (154).

A breakthrough in vaccinology has been the prevention of infection-associated cancers, for which the hepatitis B vaccine was the pioneer (155). In 1982, using molecular biology techniques, the first subunit vaccine against hepatitis B was developed. This vaccine is based on the production and purification of a surface protein from the virus and has been essential in reducing the transmission of this hepatotropic infection and preventing hepatocellular carcinomas (66, 156). This vaccine is currently part of the infant immunization regimen in most WHO member countries (133).

In the 1980s, there was significant progress in the implementation of new strategies for vaccine design. During this period, vaccines against the three main bacterial “killers” in children, *Haemophilus influenzae* type b (Hib), *Streptococcus pneumoniae* and *Neisseria meningitidis* advanced albeit with differences, using a similar strategy. The first approach was the development of capsular polysaccharides vaccines. In 1981, the strategy partially worked for *N. meningitidis* serogroups A, C, W, and Y (64), but not for serogroup B due to the molecular mimicry between the pathogen capsule of this serogroup and lipids of the human central nervous system (157, 158). In 1983, a 23-valent vaccine against *S. pneumoniae* was licensed (74). Concomitantly, in 1985, the polysaccharide vaccine against Hib was licensed (68). However, subsequent trials revealed that these polysaccharide vaccines were insufficient in eliciting adequate protection in infants (159–161).

Consequently, polysaccharide-protein conjugation strategies, originally conceived in the 1930s, were applied to enhance the immunogenicity of these vaccines (133, 162, 163). In 1987, the first Hib conjugate vaccine was licensed (69, 164). In 1999, the first *N. meningitidis* serogroup C conjugate polysaccharide (MenC) vaccine became available (73, 163, 165), and in 2005, a conjugated vaccine for serogroups A, C, W, and Y (MenACWY) was licensed (166). In 2000, the first *S. pneumoniae* conjugate vaccine (PCV) was licensed including seven serotypes (PCV7), progressing to PCV10 and PCV13, and, more recently, PCV15 and PCV20 (74).

In 1991, licensing the first inactivated oral cholera vaccine (OCV) was a significant milestone (70). This vaccine has been instrumental in controlling cholera, a diarrheal disease caused by the bacterium *Vibrio cholerae*. The OCV has been especially useful in cholera-endemic regions, during outbreaks, and emergencies, such as armed conflicts and natural disasters, where sanitation conditions may deteriorate, increasing the risk of spreading cholera (167).

A year later, in 1992, the recombinant acellular vaccine against whooping cough was developed. Providing a safer and less reactogenic alternative to the preceding whole-cell pertussis vaccine, it has since replaced the latter in many countries (71). Additionally, this year marked another milestone with the licensing of the first inactivated vaccine against hepatitis A (168), followed by the licensing of several subsequent hepatitis A vaccines (72).

### 3.6 21st-century vaccines and emerging technologies

In the 21st century, the development of new vaccines has continued to progress, leading to vaccines against rotavirus and human papillomavirus (HPV). Globally, rotavirus is the predominant cause of acute diarrhea in children under five. Two rotavirus vaccines, one based on virus attenuation and the other on the novel virus reassortment technique (allowing the expression of a specific gene in a selected animal rotavirus strain as the backbone), were licensed in 2006. These vaccines and few others that have followed have since been adopted in over 100 countries (75, 169). Responding to the significant rotavirus disease impact during childhood, the WHO recommended including an oral rotavirus vaccine in routine childhood immunization programs in 2009. As a result, countries that adopted rotavirus vaccines have reported a 40% reduction in hospitalizations due to rotavirus in children under five. At the same time, annual deaths worldwide from rotavirus-induced diarrhea have decreased by 25% (170).

A breakthrough in cancer prevention was the development of first HPV vaccine, which was licensed in 2006. This vaccine includes specific attenuated oncogenic types, and has proven to be highly effective in protecting against cervical cancer and other HPV-associated cancers in females and males (76, 171). HPV vaccines have been incorporated into immunization programs in many countries. The immunization strategy notably emphasizes the application of this vaccine in women during early adolescence. However, it is worth noting that the vaccine is also effective for men and is recommended for the prevention of anal cancer, penile cancer, and other HPV-associated cancers (171). Furthermore, it should be noted that HPV vaccines are a preventive measure, they do not serve as a cure for these cancers, nor do they protect against all types of HPV. However, they do offer protection against the most common oncogenic HPV types, which vary among different commercial vaccines (172).

The advent of reverse vaccinology (RV) has substantively modified our understanding and approaches to vaccine research, especially for the development of *N. meningitidis* serogroup B (MenB) vaccine. Unlike classical methods based on Pasteur's “isolate, inactivate, and inject” principle, RV employs whole genome sequencing (WGS) and robust bioinformatic analysis to predict the antigenic repertoire of a pathogen. This innovative approach is essential for pathogens such as MenB, for which conventional approaches have been ineffective (173).

As discussed previously, antigenic mimicry between the MenB capsular polysaccharide and human glycoproteins leads to poor immunogenic responses and raises concerns about autoimmunity (158). In 2000, the complete genome sequence of MenB MC58 was published (174). Using bioinformatics tools, a comprehensive analysis of this genome revealed 570 proteins that were predicted to be either surface-exposed or secreted. Of these, 350 were successfully cloned and expressed in *Escherichia coli*. These recombinant proteins were injected into mice, showing a promising finding, as 91 exhibited immunogenic properties and 28 triggered the production of bactericidal antibodies, suggesting their potential in vaccine development (175). The identification of these novel bactericidal antigens marked a significant advance in the field, given that only a few such antigens had been identified until then (77).

The increased availability of MenB genomes facilitated a comprehensive analysis of globally circulating MenB strains, offering insights into the diversity and conservation of meningococcal antigens. This analysis resulted in the identification of three conserved and bactericidal antigens: Neisseria Heparin Binding Antigen (NHBA), *N. meningitidis* adhesion A (NadA), and factor H binding protein (fHbp). These antigens, formulated with detergent-extracted outer membrane vesicles from a New Zealand MenB epidemic isolate, culminated in the development of the first MenB vaccine, denominated 4CMenB (176, 177). This multicomponent vaccine received approval in 2013 in Europe and Canada, and in 2015 in the United States, among other countries (77). Concurrently, a second MenB vaccine was developed, known as the rLP2086 vaccine. This vaccine, which contains two variants of the fHbp protein, was approved in the United States in 2014 and in Europe in 2017(178). In 2017, a clinical trial was initiated to evaluate the immunogenicity and safety of a pentavalent meningococcal ABCWY vaccine that combines two licensed vaccines, the MenACWY vaccine and the rLP2086 vaccine (179).

Currently, the pace of vaccine development continues to accelerate impressively, a trend fueled by the COVID-19 pandemic. This pandemic underscored the importance of centuries of accumulated knowledge in vaccinology, including technologies that had not been widely applied, but that seemed promising. As a result, an unprecedented number of different types of vaccines aimed at containing SARS-CoV-2 were developed in record time. Existing infrastructure for new vaccine platforms, such as mRNA- and DNA-based vaccines, vector-based delivery systems, as well as extensive previous work with related coronaviruses, namely SARS-CoV-1 and MERS, were critical for the rapid development of these vaccines. This previous knowledge enabled a rapid transition from preclinical evaluation to Phase I clinical trials for some of the leading vaccine candidates (180).

Among the most innovative vaccine development technologies that emerged during this pandemic are those based on mRNA, which is introduced into human cells either through viral vectors or encapsulated in liposomes. These novel vaccines have proven to be safe and effective against SARS-CoV-2 and have decisively contributed to resolving the global health emergency caused by this pathogen (181, 182). In a later section, we will delve deeper into these vaccine types.

In 2023, the first vaccines against Respiratory Syncytial Virus (RSV) were approved in the United States and Europe. The journey to develop an effective vaccine against RSV was marked by significant challenges. In the 1960s, a formalin-inactivated RSV vaccine, rather than conferring protection, exacerbated severe lung inflammatory responses during natural RSV infections in children. Consequently, safety concerns profoundly delayed RSV vaccine development for decades (21).

However, the landscape of RSV vaccine research changed due to increased understanding in the biology of this virus and its structure (183, 184). The RSV surface is decorated with proteins, including the fusion protein (F), which is a major target for vaccine development due to its essential role in viral entry and to its sequence conservation. The F protein has two complex structural conformations, the prefusion and postfusion states. The antigenic complexity and conformational dynamics of this protein underscore the intricate challenges in RSV vaccine development. Notably, prefusion F protein is present in infectious RSV but absent on the surface of formalin-inactivated RSV (185).

The first licensed RSV vaccine, denominated RSVPreF3 OA, contains the prefusion F protein and the AS01 adjuvant. This vaccine is approved for use in adults over the age of 60 (86). The second licensed RSV vaccine, denominated RSVPreF, is a bivalent vaccine containing equal amounts of the prefusion F protein from the two predominant RSV subgroups (RSV A and RSV B). This later vaccine is also approved for use in adults over the age of 60 (186), and in pregnant women between 32- and 36-weeks of gestation, to protect infants up to the age of 6 months (87).

## 4 Immunological basis of vaccination

The functionality of vaccines can only be fully appreciated by exploring some fundamental immunological concepts (see Box 2 for a summary of these key concepts).

Box 2 **Basic concepts of immunology and vaccines.**

**Antigens:** Molecules, typically proteins or polysaccharides, present on the surface of pathogens. Antigens are recognized by the immune system as foreign and trigger an immune response.

**Adjuvants:** In the context of vaccinology, they are components capable of enhancing and/or shaping antigen-specific immune responses. The use of adjuvants makes it possible to reduce the amount of antigen needed in a vaccine and improve the duration and magnitude of the immune response (187). Commonly incorporated adjuvants in human vaccines include aluminum salts, oil-in-water emulsions (such as MF59 and AS03), and bacterial derivatives (such as monophosphoryl lipid A) (188).

**Innate response:** The first line of defense of the immune system, acting quickly but lacking specificity. It involves activating cells such as macrophages, dendritic cells, and neutrophils, and, which recognize and eliminate pathogens through processes such as phagocytosis and the release of antimicrobial substances.

**Antigen presentation:** Process in which specialized cells, such as dendritic cells, capture, process, and present antigens on their surface along with major histocompatibility complex (MHC) molecules. This allows the T lymphocytes to recognize part of the antigen and subsequently become activated.

**Adaptive response:** Second line of defense of the immune system, characterized by its specificity and memory. It involves the activation of T lymphocytes and B cells in response to specific antigens, leading to a more precise and lasting immune response.

**T Lymphocytes**: Classified into two main types: CD4 and CD8. CD4 T lymphocytes, also called “helper” cells, recognize antigens presented by class II MHC molecules and aid in activating and regulating the immune response. CD8 T lymphocytes, known as “cytotoxic”, recognize antigens presented by class I MHC molecules and directly eliminate pathogen-infected cells.

**B cells:** Lymphocytes that differentiate into antibody-producing plasma cells upon being activated by an antigen. The antibodies produced are specific for the antigen that activated the B cell.

**Antibodies:** Also known as immunoglobulins, these are specialized proteins that bind to their target antigen and can directly neutralize pathogens and/or mark them to facilitate their elimination through other effector functions.

**Effector functions:** Actions performed by immune cells to eliminate pathogens and protect the organism. These functions include phagocytosis by innate cells, releasing cytokines and chemokines that promote inflammation and activation of immune cells, the production of antibodies by B cells, and elimination of infected cells by cytotoxic T lymphocytes.

**Immune memory:** Key feature of the adaptive immune system that allows for a faster and more efficient response to future exposures to the same antigen. Immune memory is due to the generation of memory B and T cells, which persist in the body after the resolution of an infection or the administration of a vaccine.

**Primary and secondary response**: Primary response is the initial immune response to an antigen, characterized by activating naïve B and T cells and producing specific antibodies. Although this response can effectively control an infection, it tends to be slower and less efficient than a secondary response. The secondary response occurs when the immune system reencounters the same antigen, and due to immune memory, memory B and T cells are rapidly activated, producing a faster, more robust, and lasting response.

The immune system is our defense mechanism against bacteria, fungi, parasites, and viruses and it has traditionally been divided into two broad components: innate and adaptive immune systems. The innate immune response serves as the first line of defense, acting quickly albeit lacking specificity. In contrast, the adaptive immune response, although slower, acts with specificity, recognizing and remembering specific pathogens to generate faster and more efficient responses upon subsequent exposures (189). Both types of immune responses actively coordinate with one another, as will be described further below.

Vaccination is possible because of adaptive immunity, with the capacity to “remember” and respond to specific pathogens. Taking advantage of this natural capacity, vaccines include the pathogen, either in live attenuated or inactivated form, or components derived from the pathogen, such as antigens or nucleic acids.

**Figure 1:**
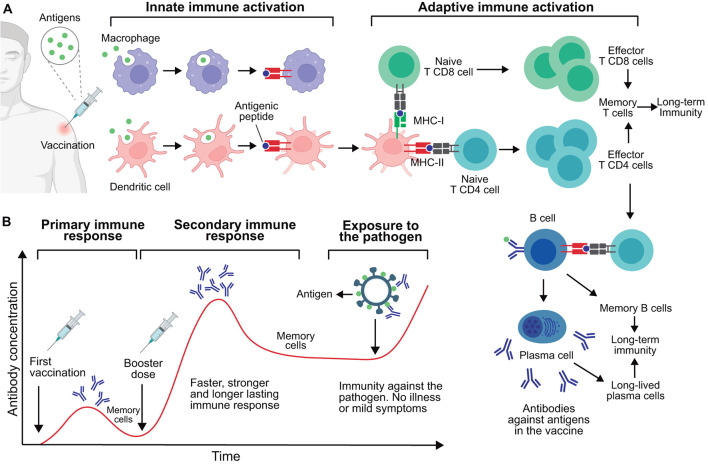
Immune response to vaccination and acquisition of immunity. **(A)** Immune response post-vaccination. This process is initiated by the activation of innate immune cells including macrophages and dendritic cells, which engulf and process antigens, leading to the presentation of antigenic peptides (epitopes) via class I or II major histocompatibility complex (MHC-I or MHC-II). These activated innate cells present antigens to CD4 and CD8 T lymphocytes, leading to their activation. Once activated, these T cells proliferate and exercise their effector functions; notably, CD4 T cells stimulate B lymphocytes specific to the antigen. These B cells proliferate and mature into plasma cells, producing antigen-specific antibodies. Of note, a number of memory T and B cells persist in the body to provide long-term immunity. Also, plasma cells can become long-lived plasma cells and secrete antibodies for months or years. **(B)** Timeline of antibody production post-vaccination. Primary and secondary immune responses are shown following the initial vaccination and subsequent booster dose, respectively. These generated antibodies and memory cells provide protective immunity against future exposure to the target pathogen. This figure was created using BioRender.com.

When the immune system encounters an antigen, either through infection or vaccination, it triggers a series of events involving several cells and molecules of the immune system (Figure 1A). A heterogeneous group of innate cells, collectively called antigen-presenting cells (APCs), including macrophages and dendritic cells, engulf the pathogen (or antigens) and present antigenically relevant structures (epitopes) on their surface to “alert” the adaptive immune system (190, 191).

T cells, important components of the adaptive immune system, recognize the epitopes presented by APCs, leading to their activation and proliferation. This generates a specialized cell population prepared to eliminate both the antigen and the corresponding pathogen. T lymphocytes are categorized into two main types: CD4 and CD8. CD4 T cells, also called helper T cells, stimulate the function of other immune system cells such as macrophages and B cells. In the case of B cells, CD4 T cells stimulate their differentiation into plasma cells, which produce and secrete antibodies. These antibodies are specialized proteins that specifically bind to antigens and aid in neutralizing or marking the pathogen for subsequent destruction by immune cells (192). CD8 T lymphocytes, also called cytotoxic T cells, can directly destroy cells infected by pathogens, thus preventing the pathogen from multiplying and spreading to other cells (193).

During this process, immune and/or infected cells release inflammatory molecules called cytokines, which are essential for coordinating the immune response. Cytokines are small proteins that serve as chemical messengers that modulate the activity of immune cells, promoting inflammation and aiding in the recruitment of additional immune cells to the site of vaccination or infection.

CD4 and CD8 T cells, B cells, antibodies, and cytokines operate synergistically to form a complex network focused on the elimination of specific pathogens and/or pathogenic molecules. Depending on the nature of the vaccine, both cellular and antibody responses can be triggered, albeit with varying degrees of potency and phenotypic differentiation. Consequently, this leads to differentiated levels of protection against specific pathogens (194).

A key feature of the adaptive immune system is immune memory. The primary immune response is triggered upon the initial encounter with a pathogen (or antigen), taking weeks to fully develop. During this response, a subset of T and B cells become memory cells that persist in the body for a prolonged period, from years to decades (195). These memory cells acquire the ability to recognize the pathogen and are quickly activated. Thus, in subsequent encounters with the same pathogen, memory cells activate rapidly, in days, triggering a secondary immune response that is faster and more efficient (196–198).

Vaccine boosters aim to induce secondary responses that enhance the immunological memory generated by the primary vaccination (Figure 1B). Typically, booster doses may increase the quantity and quality of the immune response involving memory cells. While a single vaccine dose can confer temporary protection, booster doses may extend this immunity. The need for one or more booster doses is determined in the preclinical and clinical evaluations carried out for any new vaccine candidate, as will be discussed further bellow.

## 5 Vaccine safety and protective efficacy/effectiveness assessment

**Figure 2:**
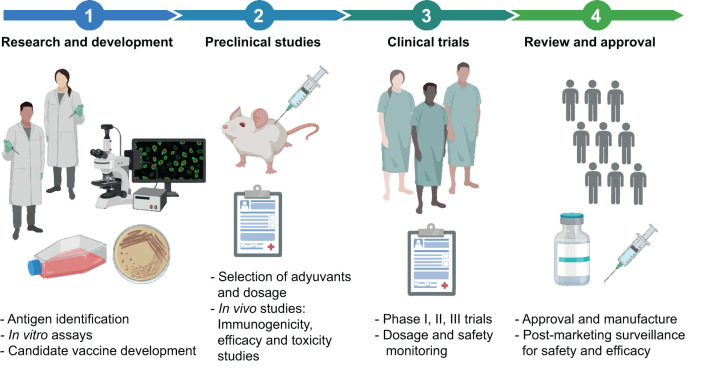
General description of the vaccine development pipeline. The process of designing, developing, and testing a vaccine involves a series of steps. It begins with the Research and development stage, where potential vaccine candidates are identified. Subsequently, preclinical studies with animals are carried out to evaluate the efficacy and safety of the vaccine candidates. The process advances to the Clinical Studies stage after a successful proof of concept of the vaccine candidate. This stage is divided into Phase I (safety and dosing), Phase II (efficacy and side effects), and Phase III (monitoring for adverse reactions in a larger population) trials. Upon successful completion of these clinical trials, the process moves to the Post-Manufacturing Approval and Phase IV, surveillance studies. Here, vaccines undergo a strict approval process to receive regulatory sanction for public use, along with ongoing surveillance to track long-term effectiveness and possible side effects. The main activities within each stage are detailed. This figure was created using BioRender.com.

The evaluation of the safety and efficacy/effectiveness of vaccines is a rigorous and meticulous process requiring both preclinical and clinical studies (Figure 2).

Before a vaccine is tested in humans, preclinical studies are performed in the laboratory, and animals, such as mice or primates, aiming to assess whether the vaccine is safe and capable of producing an effective immune response. If results obtained during this phase are promising, the vaccine can progress to clinical trials (199).

Clinical trials are studies conducted in various phases, all of which must be completed before the vaccine can be approved for public use. However, during health emergencies, such as the COVID-19 pandemic, the process can be expedited without significantly compromising safety (accepting a somewhat lower threshold for the “emergency use” restriction of these pandemic vaccines). In these situations, phases of clinical trials may overlap or be conducted simultaneously (180, 200), and regulatory agencies can advance the emergency authorizations based on interim analyses (201). It is essential to highlight that, even under expedited timelines, the risk-benefit balance is critically evaluated, ensuring that the potential benefits of vaccines used in the face of a high-impact public health crisis outweigh the potential risks.

During Phase 1 clinical trials, the vaccine is tested in a small group of people to evaluate its safety, determine the appropriate dosage, and monitor the induced immune response. Phase 2 expands the trial to hundreds of people, providing additional information on vaccine safety, its ability to generate an immune response, and a first evaluation of its protective efficacy (PE) against the main outcomes to be prevented (199, 202).

In Phase 3 trials, the vaccine is tested on thousands of people to evaluate its PE against primary and secondary outcomes and monitor side effects in a more extensive and more diverse population. Protective efficacy of a vaccine can be determined through criteria such as infection prevention and/or prevention of moderate to severe disease, including deaths if feasible (202, 203).

If the vaccine proves to be safe and effective in Phase 3 clinical trials, health regulatory entities, such as the United States Food and Drug Administration (FDA), the European Medicines Agency (EMA), and others, can proceed to its approval, an essential step for vaccine licensing and use.

Once approved and distributed, the vaccine enters what is known as Phase 4 evaluations, or post-marketing surveillance (a term coined for non-case-control trials). During this stage, the safety and effectiveness of the vaccine continue to be evaluated in a real-world setting, with broader and more diverse population tracking. Phase 4 enables the collection of long-term data on the efficacy of vaccines, their effects on disease incidence, hospitalizations, and fatalities in various age groups and health conditions. It also allows monitoring for unforeseen and/or rare adverse effects that may arise when the vaccine is used in a much larger and diverse group of people (202).

Adverse effects, which both healthcare professionals and vaccinated individuals can report, are recorded, and carefully analyzed. These reports are vital for ensuring the ongoing safety of the vaccine and allow regulators and vaccine manufacturers to quickly detect and respond to any safety signal that may arise.

It is important to note that vaccine efficacy/effectiveness can be influenced by various factors such as the endogenous microbiota, genetic traits, age, and nutritional status of the individual, presence of chronic or immunosuppressive disease, among others (204). These factors must be considered when designing and implementing vaccination programs to ensure optimal safety and protection of the population.

## 6 Types of vaccines

**Figure 3:**
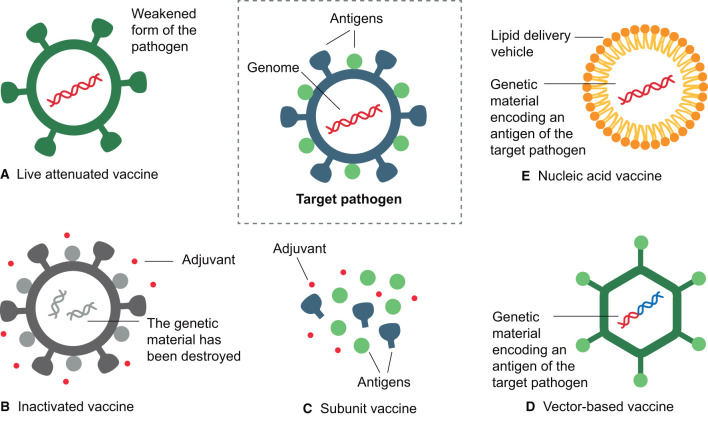
Main types of vaccines. **(A)** Live attenuated vaccines use a weakened form of the pathogen. **(B)** Inactivated vaccines contain a killed version of the pathogen, with surface antigens intact but the genome inactivated. **(C)** Subunit vaccines include only selected antigens from the pathogen. **(D)** Vector-based vaccines use a harmless vector to carry a fragment of the genome of the target pathogen. **(E)** Nucleic acid vaccines use the genetic material from the pathogen, either DNA or RNA, encapsulated within a delivery mechanism, such as liposomes or introduced through electroporation (DNA). All these types of vaccines aim to stimulate the immune response to a specific pathogen, although they may have different mechanisms of action. This figure was created using BioRender.com.

In this section, we will explore the different types of vaccines (Figure 3), their main characteristics, advantages, and limitations (Table 2). From attenuated vaccines that use weakened pathogens to nucleic acid vaccines that encode specific antigens, vaccine design has evolved with advancing technology to improve safety, efficacy, production efficiency, and stability.

**Table 2:** Main characteristics, limitations, and disadvantages of available vaccines.

**Type of vaccine**	**Characteristics**	**Limitations and disadvantages**
Live attenuated	Weakened version of the pathogen. Provides durable immunity, often with a single dose. Although for several vaccines, repeated doses are also required.	Not recommended for immunocompromised individuals. Small risk of the pathogen reverting to its virulent form.
Inactivated	Inactivated pathogens, which cannot replicate, ensure safety even for individuals with compromised immune systems.	Requires multiple doses. Protection tends to be less durable than live attenuated vaccines.
Subunits	Purified parts (antigens) of the pathogen. Safe for immunocompromised individuals.	Requires multiple doses. Protection tends to be less durable than live attenuated vaccines.
Toxoids	Non-toxic derivatives of toxins (toxoids). Triggers an immune response against the toxin, not the pathogen itself.	Requires multiple doses. Some individuals may have allergic reactions to the toxins.
Vector-based	Carrier microorganism (vector) transporting genetic sequences encoding for a relevant antigenic protein of the target pathogen. The vector may or may not be replicative.	Potentially reduced efficacy among individuals with pre-existing immunity to the vector. Replicative vectors are not suitable for immunocompromised individuals.
Nucleic acids	Genetic material (mRNA and, less commonly, DNA) encoding a relevant virulence protein, which is encapsulated in a lipid vesicle or introduced by electroporation. Can be rapidly developed and produced.	Requires extremely low storage temperatures. Long-term effects under study.

### 6.1 Live attenuated vaccines

These vaccines employ microorganisms weakened through various processes, such as serial passage in cell cultures or unconventional hosts. Essentially, by continually propagating the pathogen in an atypical environment, the microorganism accumulates genetic mutations and/or loses virulence genes, leading to its attenuation and therefore its ability to cause disease in the original host. Additionally, advancements in genetic engineering have provided faster and more reliable methodologies to delete or modify genes with the aim of attenuation (205).

However, attenuating a pathogen to produce a vaccine can be complicated and expensive, being especially challenging for bacteria, structurally more complex than viruses, with a larger number of genes and various virulence mechanisms. As a result, few live attenuated bacterial vaccines are commercially available (206).

Attenuation allows the pathogens to retain their ability to replicate in the host, allowing them to mimic a natural infection to some extent but without causing the disease. This characteristic allows these vaccines to induce a comprehensive and long-lasting immune response, generating both humoral and cell-mediated immunity (205).

Prominent examples of live attenuated vaccines include vaccines for tuberculosis (BCG), poliovirus (OPV), measles, mumps, and rubella viruses (MMR), rotavirus, and yellow fever (205). These vaccines are generally safe and effective; however, they may present risks under specific circumstances. The attenuated pathogen could potentially cause disease or adverse effects for immunocompromised individuals or pregnant women. Also, although extremely rare, there is a chance that the attenuated pathogen could revert to a virulent form and cause disease (207).

Limitations of these vaccines compared to other types of vaccines include lower stability with a shorter shelf life often requiring refrigeration, which can complicate storage and transport, particularly in resource-limited regions (208).

### 6.2 Inactivated vaccines

Inactivated vaccines, also referred “killed vaccines”, are among the earliest vaccines developed. These vaccines are manufactured from microorganisms that, after being subjected to chemical or physical treatments, lose their ability to replicate, thus eliminating their potential to cause disease in any host. Despite inactivation, the remaining pathogen structures retain the ability to be recognized by the immune system, triggering an immune response, most commonly humoral, thereby conferring immunity (209, 210).

Inactivation can be achieved through chemical or physical processes. In the former, agents such as formaldehyde/formalin or β-propiolactone are used. Formalin generates cross-links between amino acid molecules, a process known as fixation. This process can stabilize the three-dimensional structure of the proteins, conserving their conformation but abolishing their biological functions. Additionally, these chemical agents can damage the integrity of nucleic acids, rendering the pathogen unable to replicate (209, 211). Physical inactivation can be achieved by heat, often at high temperatures (>60°C). However, this approach is frequently accompanied by a chemical treatment to ensure thorough pathogen inactivation (209, 212).

Inactivated vaccines have several advantages. They are safe and well-tolerated, even among immunocompromised individuals or pregnant women, as the inactivated pathogen cannot replicate or revert to a virulent form (213). Additionally, they are economically feasible and relatively straightforward to produce.

However, they also have limitations. Inactivation methods can eventually alter the structure of some relevant antigens, reducing the neutralizing capacity of induced antibodies. Moreover, as they do not mimic a natural infection, the immune response may be of shorter duration and magnitude compared to attenuated vaccines. Repeated booster doses are usually required to maintain long-term protection. Additionally, the majority of these vaccines require the incorporation of adjuvants to increase immunogenicity (211). Advances in new adjuvants, for which extensive developments have occurred in the past decades, improve the effectiveness of these vaccines (214, 215).

Among the potential risks associated with inactivated vaccines is the possibility of incomplete pathogen inactivation, which could cause post-vaccination outbreaks. Although this situation has occurred, current rigorous regulations and stringent quality controls have substantially reduced this risk (216).

Prominent examples of inactivated vaccines include vaccines for poliovirus (IPV), hepatitis (HepA), influenza, and rabies (211, 217). In addition, inactivated whole-cell vaccines have been used for bacterial diseases, such as pertussis (whooping cough) and cholera (167, 210). In the recent COVID-19 pandemic, several inactivated vaccines against SARS-CoV-2 were developed (78–80).

### 6.3 Subunit and conjugate vaccines

These vaccines contain only specific fragments (subunits) of the pathogen they are intended to protect against, rather than the entire pathogen. The subunits can be peptides, proteins, or polysaccharides derived from the pathogen. Although not infectious, these subunits are still capable of triggering an immune response; in other words, they are immunogenic (218).

Developing these vaccines requires identifying, producing, and purifying the antigenic components of the pathogen that can induce an effective protective immune response. In this process, the nature of the antigen used is a key factor. For instance, protein antigens tend to be more potent immunogens than polysaccharides, triggering responses from both B and T cells (207). An example is the hepatitis B vaccine, which employs a protein from the surface of the virus as a subunit (156). Another example is the acellular pertussis vaccine, which uses several purified proteins from *B. pertussis* (219).

In contrast, polysaccharide subunit vaccines induce B cell responses, albeit they typically do not activate T cells, nor do they usually generate immunological memory. Therefore, conjugate vaccines have been developed to enhance the immunogenicity of polysaccharide antigens. This approach links a polysaccharide to a carrier protein, allowing a more effective T cell response. This method increases the immunogenicity of polysaccharides, especially in infants < 2 years of age. Polysaccharide-protein conjugation allows the immune system to recognize and respond more effectively, producing polysaccharide-specific antibodies and generating memory cells (219). The pneumococcal, meningococcal, and *H. influenza* type b conjugate vaccines are successful examples of this type of vaccine (220).

Subunit vaccines present several advantages. They are generally safe and well-tolerated, given that they lack live microorganisms that can cause disease. Furthermore, their high specificity generates a more targeted immune response, thereby circumventing potential adverse effects of a broader immune response (more intense inflammation, fever, malaise, among others). Production of these vaccines is straightforward and adaptable, and their lyophilization facilitates transport and storage without the need for refrigeration (221, 222).

Subunit vaccines are not without challenges. Although they are less reactogenic, their ability to stimulate robust and lasting immune responses is usually inferior to that of attenuated vaccines, more similar to inactivated vaccines. Thus, adjuvants and multiple doses are often required to achieve a long-term protective response (221, 222).

Furthermore, developing these vaccines requires a deep understanding of the components of the pathogen that trigger protective immunity, as well as an understanding of the immune responses necessary to protect against specific pathogens. This knowledge guides the choice of the antigenic components to be incorporated into the vaccine and the methods required to evaluate immunogenicity (207, 218). This can be challenging, as promising results in preclinical trials do not always translate into success in clinical trials due to various factors, including variability in immune responses between different species and the possible insufficiency of adjuvant potency (218).

### 6.4 Toxoid vaccines

Inactivated bacterial toxins are called toxoids. In general, the manufacturing process of these vaccines involves bacterial culture in a laboratory environment, purification, and inactivation of the toxin with formalin or another chemical agent. This inactivation aims to eliminate toxicity while preserving the ability to induce a specific immune response against the toxin (223).

Once the vaccine is administered, the immune system identifies the toxoid as a foreign antigen and produces specific antibodies called antitoxins. Consequently, in the event of future exposure to this toxin-producing bacteria, these antitoxins can neutralize the toxins, preventing damage to cells and tissues (224). Toxoid vaccines do not contain live microorganisms and thus cannot revert to virulent forms. However, these vaccines may also require adjuvants and booster doses to maintain long-term protection, as the immunity may decrease over time (223).

Classic examples of toxoid vaccines include vaccines against diphtheria and tetanus. These are often administered in combination with the pertussis vaccine in the combined DTP and DTaP (diphtheria, tetanus, and acellular pertussis) vaccines (225, 226), a more recently in the hexavalent DTaP5-IPV-Hib-HepB vaccine (227).

### 6.5 Vector-based vaccines

These vaccines are a recent breakthrough in vaccinology, based on the use of no pathogenic microorganisms, known as vectors, acting as a “Trojan horse”. Genetic engineering techniques modify these vectors, incorporating a DNA or mRNA fragment that encodes for a specific antigen from a pathogen. Thus, the vector can express this genetic material and produce the desired antigen within host cells, leading to its recognition by the immune system (228, 229).

Prominent viral vectors currently in use include adenovirus, measles virus, influenza virus, and poxvirus. These vectors can be replicative (attenuated) or can be genetically modified to be non-replicative (inactivated), a measure that enhances the safety profile of these vaccines (81).

The development of vector-based vaccines has challenges, as the genetic manipulation of the vectors requires a high degree of precision and control to ensure the safety and effectiveness of the vaccine. Additionally, pre-existing immunity to the vector within the population or provided by primary vaccination could potentially compromise vaccine efficacy/effectiveness (82).

Before the COVID-19 pandemic, the licensure of vector-based vaccines was limited to ebola virus (83). However, the pandemic required a rapid response that led to the development of several vaccines based on viral vectors that express the SARS-CoV-2 spike protein. These include the ChAdOx1 vaccine, which uses a modified chimpanzee adenovirus (84); the Ad26.COV2-S vaccine, which uses a type 26 adenovirus (85); the Sputnik V vaccine, which uses two adenoviral vectors, type 26 (prime) and type 5 (booster); and the Ad5-nCOV vaccine, which uses adenovirus type 5 (78, 228).

Recently, vector-based vaccines against RSV have also been developed, which are under clinical evaluation with promising results. These include the Ad26.RSV.preF vaccine, with a recombinant adenovirus serotype 26 vector encoding the prefusion F protein (230), and the MVA-BN RSV vaccine, with a modified vaccinia Ankara virus vector encoding Ga, Gb, F, and M2 proteins (231).

The mechanism of action of these vaccines is genuinely innovative. Taking the ChAdOx1 vaccine as an example, the genetically modified adenovirus (vector) enters the cell, transporting the Spike protein gene into the cell nucleus of various host cells. This gene is then transcribed into mRNA, which subsequently migrates to the cytoplasm. Within the cytoplasm, the ribosomes use the mRNA as a template to produce the Spike protein. Once produced, this protein is presented to the immune system, triggering an immune response against SARS-CoV-2 (228).

The successful outcome of vector-based vaccines during the pandemic suggests that they may play an increasingly pivotal role in the future. Their ability to generate robust and long-lasting immune responses, added to the versatility to be adapted against a variety of viral infections, establishes these vaccines as a powerful and relevant tool in vaccinology.

### 6.6 Nucleic acid vaccines

Nucleic acid vaccines will most likely become a turning point in vaccinology. Like vector-based vaccines, nucleic acid vaccines use DNA or RNA molecules that encode for pathogen-specific antigenic proteins. The former use a plasmid as the vehicle for the genetic material, while the latter have mostly used encapsulation in lipid nanoparticles (232, 233).

There are two categories of nucleic acid vaccines: DNA and RNA. When a DNA vaccine is administered, mainly through electroporation, the DNA enters host cells and is transported to the nucleus, where it is transcribed into mRNA. The mRNA is transported out of the nucleus, to the ribosomes responsible for synthesizing the desired antigen. This antigen undergoes processing and presentation to immune cells, thus eliciting a specific immune response (234). Unlike DNA vaccines, RNA vaccines allow direct translation of the antigen within the cytoplasm. As with DNA vaccines, the result is a specific immune response against the target pathogen (235).

This technology has been particularly relevant in the context of the COVID-19 pandemic (236). The BNT162b2 and the mRNA-1273 vaccines are notable examples of mRNA-based vaccines encoding the spike protein (237). In light of their safety and efficacy, they received emergency use authorizations and approvals in numerous countries, enabling the implementation of widespread vaccination (236). Importantly, these mRNA vaccines have demonstrated over 90% efficacy in preventing symptomatic COVID-19 disease in clinical trials. Most important, they proved to provide significant protection against severe forms of the disease and hospitalizations (238).

Nucleic acid vaccines are a versatile platform offering flexibility in design and scalability in production. Due to its adaptability, it is feasible to adjust the genetic sequence of the antigen, which allows the rapid adaptation of vaccines to new variants of the pathogen. This prompt adjustment could potentially enhance the accuracy and efficacy of the immune response against the circulating variants (239). This platform could also be employed to design vaccines against multiple pathogens (240, 241).

Limitation of mRNA vaccines include the fragile nature of the mRNA, prompting the need for cold storage at exceedingly low temperatures to maintain their stability, which can represent significant logistical challenges, especially in underdeveloped regions (238). Additionally, although rare, allergic reactions to mRNA vaccines have been reported (242), as well as uncommon severe side effects such as Bell's palsy (243), Guillain Barré syndrome (244), and myocarditis/pericarditis (245).

Beyond vaccines, mRNA technology is also being implemented for a variety of other medical applications, such as gene therapy and immunotherapy for the treatment of genetic diseases and cancer, respectively. These applications reflect the broad potential of mRNA-based therapeutics in the near future (246).

## 7 Public health and economic impacts of vaccination

Health professionals and biomedical researchers tend to measure the benefits of vaccines in terms of disease prevention and mortality reduction. However, it is also important to recognize and quantify the economic and social benefits of vaccines and immunization programs at both the individual and community levels. It is equally important to effectively communicate these benefits to the general public and policymakers to promote vaccination acceptance, increase immunization coverage, and encourage investments in novel vaccine development (247). In this section, we will briefly examine the impact of vaccines on public health and their economic and social benefits.

### 7.1 The public health value of vaccination

The most significant impact of vaccines has been their role in decreasing morbidity and mortality caused by infectious diseases that in the past were disabling or fatal (248). People today live more and better due to the control of threatening infections. For instance, in the United States, a historical comparative study by Roush et al. (118) highlighted the transformative impact of immunization on the incidence of infectious diseases. This research analyzed morbidity and mortality data associated with 13 vaccine-preventable diseases (VPDs), demonstrating a reduction of over 90% following the implementation of vaccination programs compared to rates before these programs were established. This remarkable achievement was possible due to high coverage for vaccines such as polio, DTaP, and MMR (247).

**Figure 4:**
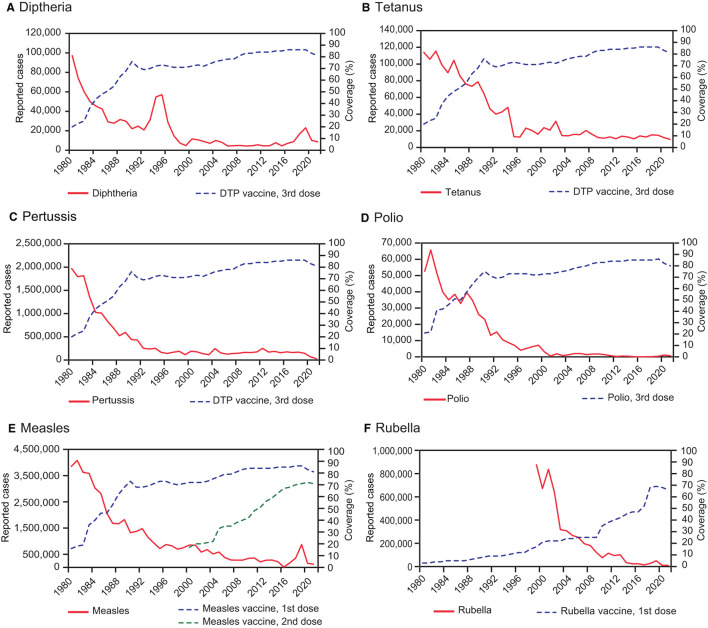
Global impact of vaccination on selected infectious diseases (1980–2021). This figure illustrates the number of reported cases for selected vaccine-preventable diseases (VPDs), including Diphtheria **(A)**, Tetanus **(B)**, Pertussis **(C)**, Polio **(D)**, Measles **(E)**, and Rubella **(F)**, from 1980 through 2021. The data was submitted to the World Health Organization (WHO) annually via the WHO/UNICEF Joint Reporting Form on Immunization (JRF). The most recent WHO/UNICEF Estimates of National Immunization Coverage (WUENIC) for these specific VPDs on a global scale are presented. Notably, the increase in vaccination coverage led to a marked decrease in the number of cases reported annually for each of these diseases. Data were sourced from the World Health Organization's immunization data portal, accessible at: https://immunizationdata.who.int/.

Vaccine distribution poses a considerable challenge in low- and middle-income countries (LMICs). Nevertheless, over the past 40 years, the increase in global vaccination rates has led to a significant decrease in the number of annually reported cases of VPDs. Figure 4 shows the worldwide impact of vaccination on select VPDs from 1980 to 2021.

Current vaccines are an efficient tool for preventing diseases related to climate change, such as cholera, yellow fever, and dengue. These diseases are expanding to new regions of the world due to floods, temperature fluctuations, or changes in disease vectors (e.g., mosquitoes) (167, 249). Alongside other public health strategies, vaccines have played a key role in controlling outbreaks, epidemics, and pandemics. Examples include the cholera epidemic in Haiti from 2010 to 2019 (250), the ebola epidemic in the Democratic Republic of Congo from 2018 to 2019 (251), and the recent COVID-19 pandemic (236, 252).

In the current public health landscape, many diseases caused by pathogenic bacteria can be prevented with vaccines. This prevention strategy reduces the need for antibiotics, thereby decreasing the selective pressure that leads to the development of resistance to these drugs. This is critical to address the growing threat of multidrug resistance in bacteria, which could be responsible for future pandemics (253, 254).

Notably, vaccines can prevent diseases beyond the specific pathogen for which they were designed. Infections, particularly caused by viruses, can predispose to secondary bacterial infections. For instance, influenza virus infection often leads to complications like bacterial pneumonia and acute otitis media (AOM) (255, 256). Indeed, vaccination against influenza can result in a modest yet significant reduction in AOM cases (257). Another noteworthy example is the impact of the introduction of the measles vaccine in the 1960s, which led to a significant reduction in child morbidity and mortality, not only associated with measles, but also with other diseases (258, 259). Measles causes immunosuppression, increasing susceptibility to secondary bacterial infections for several weeks to months, particularly those caused by *S. pneumoniae* and Hib (259, 260). Thus, measles vaccination has been proposed as a preventive measure against these secondary bacterial infections (258, 261).

The scope of vaccines goes beyond the prevention of diseases at the individual level, as they also protect communities through herd or collective immunity. When a significant portion of the population acquires immunity against a pathogen that is readily transmissible from person to person, either through vaccination or by having overcome the infection, the spread of the pathogen decreases considerably. This protects even those who cannot receive the vaccine due to age or medical conditions. This indirect protection is especially crucial for safeguarding vulnerable individuals, such as newborns, older adults, and people with weakened immune systems (262, 263).

### 7.2 Economic and social benefits of vaccination

Vaccines, beyond their direct impact on health, offer substantial economic benefits and contribute to poverty reduction. In many LMICs, where healthcare coverage often remains inadequate, people commonly must face high out-of-pocket (OOP) medical expenses. Econometric studies estimate that increasing vaccination coverage in LMICs can save billions in OOP expenses, thus preventing millions of people from facing catastrophic health expenses. These are defined as a significant proportion (usually 10–25%) of household income or expenditures (264). Consequently, by preventing disease, vaccines represent a cost-effective strategy that mitigates the financial burden on both families and health systems. This reduction in expense is seen through the avoidance of costly and time-consuming medical tests, procedures, and treatments.

Vaccines also play an important role in mitigating productivity losses associated with absenteeism and presenteeism (265). Absenteeism refers to instances where employees are unable to work due to illness. On the other hand, presenteeism reflects a scenario where employees continue to work while sick, resulting in suboptimal productivity levels due to illness-related impairments. By preventing disease, vaccinations can enhance overall workforce productivity, whether employees operate in traditional office settings or from remote environments, thereby stimulating economic growth. Moreover, reducing childhood disease incidence decreases parental absenteeism, as parents would otherwise need to take days off to care for their sick children. This dynamic has a significant economic impact, further underscoring the comprehensive value of vaccination (266).

The socio-educational benefits of childhood vaccination merit emphasis. Vaccination allows children to attend school and participate in community activities without interruption from debilitating diseases (267). Studies conducted in LMICs reveal that childhood vaccination, by preventing diseases, can boost physical and cognitive development, improve educational performance, and increase lifetime earnings.

Such studies consistently associate childhood vaccination with an additional 0.2–0.3 years of education in various countries. This impact is even more evident in economically disadvantaged groups, highlighting the social and economic value of childhood vaccination (268).

In this context, vaccines are a tool, in universal programs, that promote equity and social benefits in healthcare. By mitigating the burden of infectious diseases that disproportionately affect the most vulnerable, vaccines enhance the quality of life and healthcare accessibility for everyone, regardless of their economic or social situation (269, 270).

The Expanded Program on Immunization (EPI), implemented in 1974 as a WHO initiative, is an example of how vaccines can reduce healthcare disparities. This initiative increased vaccination coverage in developing countries from 5 to 80%, significantly improving children's life opportunities and health equity (270).

Finally, vaccines promote a safer and more efficient exchange of people and goods internationally by contributing to controlling outbreaks. This effect drives trade and tourism, which in turn promotes economic growth (248, 271). Thus, vaccines play a key role not only in individual and collective health, but also in global social and economic development.

## 8 Origin, impact, and mitigation of vaccine hesitancy

Vaccine hesitancy is characterized by a delay in acceptance or outright refusal to vaccines despite the availability of vaccination services (272). Several models have been proposed to elucidate the nature of vaccine hesitancy. For instance, the “Three C's” model proposed by MacDonald et al. (273), identified complacency, convenience, and confidence as influential factors. Additionally, Hagood and Herlihy (274), classified individuals into four groups: vaccine-acceptor, vaccine-hesitant, vaccine-resisting, and vaccine-rejecting. Meanwhile, the Sage Working Group proposed the Vaccine Hesitancy Continuum, which describes a spectrum ranging from unconditional acceptance of all vaccines to complete refusal. Individuals who are vaccine-hesitant fall somewhere in between these two extremes, forming a diverse group (272). It is important to note that while these classifications provide valuable insight into the various attitudes toward vaccination, they will not be used explicitly in this review. However, recognizing this spectrum of vaccine-hesitant individuals is important to understanding this phenomenon.

### 8.1 Origin of vaccine hesitancy

The phenomenon of vaccine hesitancy has been present since the introduction of vaccination. Its history, as old as that of vaccines themselves, is marked by persistent resistance through several milestones in medical advancement. For a comprehensive historical analysis of vaccine hesitancy, the reader is referred to previous extensive reviews (275–277).

The origin of vaccine hesitancy date back to the late 18th century. The introduction of the smallpox vaccine by Jenner in 1796 elicited both admiration and criticism. As discussed in a preceding section, Jenner inoculated individuals with material from cowpox lesions, raising fears and misconceptions (47). This unfamiliar method, combined with religious beliefs and distrust in medicine, planted the initial seeds of hesitation. Some individuals feared that the procedure would lead to “bovine” characteristics in humans, while others believed it went against God's will (31, 278).

Despite these concerns, the effectiveness of the smallpox vaccine was undeniable, leading to its rapid adoption and spread throughout Europe and the United States. Nonetheless, a segment of the population consistently opposed vaccination. In the mid-19th century, some Western countries instituted mandatory vaccination laws, imposing stringent penalties for non-compliance, to safeguard public health (279).

These mandatory vaccination policies often met with public opposition, being perceived as violations of personal freedoms, and gave rise to anti-vaccine groups and major legal battles (280). These groups, later termed as “Anti-vaxxers” in contemporary discourse, were driven by a variety of factors ranging from concerns about vaccine safety and efficacy to broader socio-political motivations (277). One of the most notable of these legal confrontations reached in the United States Supreme Court in 1905. In a landmark judgment, the court reaffirmed the authority of the state to mandate vaccinations to protect the public from communicable diseases (281).

The 20th century saw an increase in both the number of available vaccines and the intensity of opposition. In the United Kingdom and the United States between the 1960s and 1980s, concerns emerged regarding potential adverse effects and neurological complications associated with the DTP vaccine. Although initial studies suggested potential risks, subsequent research refuted any link between the vaccine and neurological damage (117, 280). Nonetheless, public skepticism led to decreased vaccination rates, resulting in disease outbreaks in numerous countries (282–284).

In more recent times, the infamous and now discredited 1998 study linking the MMR vaccine to autism stands out as the best example of the impact of misinformation (285, 286). The extensive media coverage of this study, even after its retraction, left a lasting mark on public perception, reducing MMR vaccination rates and leading to measles outbreaks in many parts of the world (287). This incident underscores the enduring effects of misinformation on public health.

A focal point in vaccine hesitancy has been concerns related to the safety of additives, or excipients, in vaccine formulations. These additives include a range of substances that enhance the immune response (adjuvants), stabilize (stabilizers) and preserve the vaccine (preservatives) (288). Critics argue that these substances, potentially harmful in large doses, pose health risk when included in vaccines. Nevertheless, scientific research has consistently demonstrated the safety of these additives in the trace amounts used in vaccines (289–291). The removal of thimerosal, a mercury-containing compound, from most vaccines in Europe in 1992 and in the United States in 2001 exemplifies the evolution of vaccine technology and regulations in response to public concerns (286, 292).

### 8.2 Vaccine hesitancy in digital era

In the digital era, the internet and social networks have revolutionized information dissemination and consumption, profoundly impacting public health communication, particularly regarding vaccine acceptance (275). The easy access to a broad spectrum of information has empowered individuals to seek health-related knowledge. However, it has also facilitated the rapid proliferation of both accurate and inaccurate information. Specifically, social networks have become hubs for spreading misinformation and creating echo chambers, where individuals predominantly encounter information that reinforces their pre-existing beliefs (293). This dynamic has significantly contributed to vaccine resistance, as misinformation about vaccine safety and efficacy can spread widely, be amplified, and prove resistant to correction (294).

The COVID-19 pandemic exemplifies these challenges. Vaccines against SARS-CoV-2 were developed, tested, and approved at an unprecedented pace, attracting attention and scrutiny. These rapid vaccine developments resulted from a global effort and substantial resource allocation, all while maintaining rigorous vaccine development standards. The COVID-19 vaccine clinical trials were conducted with a meticulous risk-benefit balance, involving overlapping or consecutive phases, guaranteeing the safety and effectiveness of the vaccines (180).

Nonetheless, the accelerated pace of vaccine development generated misconceptions and hesitancy, contributing to an “infodemic” characterized by an overwhelming flood of both information and disinformation across various media channels. Social media platforms played a central role in disseminating both accurate information and misinformation, leading to public confusion and skepticism (295). The predominant reasons for refusing COVID-19 vaccines included general opposition to vaccines, concerns about the safety of rapidly developed vaccines, potential unknown short- and long-term adverse effects, and perceptions of COVID-19 as being relatively harmless (296). Notably, these claims have been actively debated and refuted with clinical and experimental evidence, highlighting the safety and protective efficacy of vaccines against severe COVID disease (see previous sections).

### 8.3 Impact of vaccine hesitancy

The consequences of vaccine hesitancy are multiple, serving to undermine the public health benefits and economic benefits associated with vaccines, which were discussed in the previous section.

From the perspective of public health, such hesitancy affects vaccine coverage, which can directly lead to the resurgence of diseases that are preventable through vaccination. This situation poses a risk not only to unvaccinated individuals but also jeopardizes herd immunity, thereby endangering communities at large. In 2019, for instance, a decline in MMR vaccine coverage, attributed to vaccine hesitancy, resulted in a resurgence of measles in numerous high-income countries (297). Furthermore, unvaccinated children face an elevated risk of contracting diseases that vaccines can prevent and may experience severe complications associated with these diseases. Glanz et al. (298) conducted a study demonstrating that children who were delayed in receiving one or more doses of the DTaP vaccine were 4.4 times (2.23–8.55) more likely to be diagnosed with pertussis compared to their peers who were vaccinated in accordance with the recommended schedule.

From an economic standpoint, outbreaks and resurgences of vaccine-preventable diseases put pressure on vulnerable families and health systems. These situations also redirect essential resources away from other critical health services (299).

### 8.4 Mitigation of vaccine hesitancy

**Figure 5:**
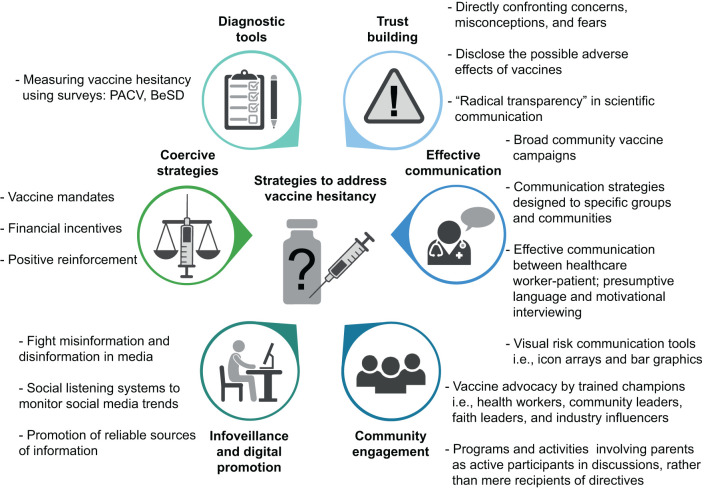
Multifaceted approach to mitigate vaccine hesitancy. Diagnostic tools, such as surveys, can be utilized to identify potential barriers to vaccination. Building trust necessitates an active approach to addressing public concerns, misconceptions, and fears, while advocating for “radical transparency” in science communication. To ensure effective communication about vaccines, comprehensive campaigns targeting both the general population and specific groups and communities are imperative. Enhancing communication between healthcare workers and patients is key, requiring the adoption of presumptive language and motivational interviewing techniques to build trust and facilitate informed decision-making. Vaccine advocates, including community leaders and healthcare workers, play a crucial role. Programs that engage parents as active participants are equally significant. In the digital age, infoveillance is crucial for monitor trends on social media platforms and counteracting misinformation and disinformation about vaccines. Lastly, while coercive strategies, such as vaccination mandates and financial incentives can be effective, their implementation must be judiciously considered and culturally and regionally adapted. PACV, parent attitudes about childhood vaccines. BeSD, behavioral and social drivers of vaccine uptake model. This figure was created using BioRender.com.

Addressing vaccine hesitancy requires a comprehensive, evidence-based approach that incorporates a variety of strategies (Figure 5). This process begins with clearly defining the extent of vaccine hesitancy, distinguishing it from other factors that may cause people to be unvaccinated or under-vaccinated. It is important to differentiate hesitancy from other barriers to vaccination, such as access issues or lack of awareness. Understanding this distinction is key to determining whether interventions specifically targeting vaccine hesitancy are required to enhance vaccine uptake rates (272).

Following this initial clarification, it is essential to identify the causes of vaccine hesitancy and thus implement programs designed to effectively address these barriers. Diagnostic tools, such as the Parent Attitudes about Childhood Vaccines (PACV) (300) survey and the Behavioral and Social Drivers (BeSD) of Vaccine Uptake model (301, 302), can be employed to assess vaccine acceptance and identify potential barriers.

Developing targeted interventions to mitigate vaccine hesitancy has key components, including building trust, providing accurate and understandable information, and actively engaging with communities. These and other strategies will be discussed below.

Directly confronting concerns, misconceptions, and fears is crucial for fostering trust. Transparency in scientific communication is of paramount importance. The rapid development and approbation of COVID-19 vaccines underscored the need for a “radical transparency” approach in vaccine communication. Transparency, even when disclosing potential negative aspects of vaccines, fosters trust in health authorities, despite potentially impacting vaccine acceptance negatively in the short term. A recent study by Petersen et al. (303), showed that transparent communication of negative vaccine information enhances trust in health authorities. Conversely, vague, and overly reassuring communication strategies fail to increase vaccine acceptance and, in fact, result in diminished trust and increased endorsement of conspiracy theories.

Communication approaches include broad community vaccine campaigns and tailored communication programs designed for specific cultural groups and communities (304). The role of effective communication between healthcare workers and patients, using techniques such as presumptive language and motivational interviewing, cannot be underestimated (305). Furthermore, risk communication tools, including visual aids like icon arrays, bar graphics, and images, enhance health literacy and support informed decision-making (306, 307).

Community engagement plays a key role in this process. Trained vaccine champions, such as health workers, community leaders, faith leaders, and industry influencers, can provide clear, transparent, and consistent information, share personal positive vaccination experiences, and act as influential role models (308–310). These individuals, by actively participating in community dialogues and addressing questions and concerns empathetically and respectfully, contribute significantly to building trust and supporting vaccination within their communities. Activities and programs that actively involve parents in discussions and decision-making about vaccines, rather than merely being recipients of directives, further promote vaccine acceptance (311).

In the digital era, fight misinformation and disinformation require the implementation of social listening systems or infoveillance. These systems monitor social media channels for emerging trends, enabling the timely identification and address of biased or non-evidence-based information before it gains widespread traction (312). Complementing these systems, it is imperative to ensure that accurate and reliable information is consistently accessible to the public (306).

Although coercive strategies, such as financial incentives, positive reinforcement, and vaccine mandates, have proven effective in increasing vaccination rates in certain contexts (313, 314), their application requires careful consideration of cultural and regional nuances (315, 316).

## 9 Conclusions and perspectives

Since the development of the first vaccine against smallpox, vaccines have emerged as one of the most effective strategies in preventing infectious diseases and promoting public health globally. Through vaccination, pathogens such as smallpox virus and wild poliovirus type 2 and 3 have been eradicated, and many others controlled, several of which are close to eradication.

The development of a myriad of vaccine platforms, each with specific advantages and limitations, has allowed us to prevent infections caused by a wide range of pathogens and protect different target populations. The COVID-19 pandemic has catalyzed rapid progress in vaccinology, culminating in the development and approval of an array of vaccines, including several based on novel technologies, in less than a year.

Looking ahead, vaccine research is expected to advance in several directions. First, current vaccine platforms will likely be refined to improve their efficacy, safety, and responsiveness to different pathogens and populations. Adjuvants will continue to be refined to enhance the immunogenicity of inactivated and subunit vaccines (215).

Second, the development of mRNA vaccines for a broad range of pathogens beyond SARS-CoV-2 is anticipated. Its rapid, scalable, and adaptable production make it a breakthrough technology that could aid in controlling neglected, emerging, and re-emerging infectious diseases (317, 318).

Third, progress in immunology and a deeper understanding of host factors influencing immunity development, such as comorbidities, nutrition, and the microbiota, are expected to yield insights into the mechanisms driving vaccine effectiveness (319, 320). This knowledge could be used to design more precise and personalized vaccines.

Fourth, innovations in vaccine delivery technology could improve the efficacy and acceptance of vaccines. For instance, novel administration methods, such as microneedle patches or intranasal delivery, could simplify vaccination and enhance the immune response compared to traditional intramuscular injection. Additionally, these methods could reduce pain and anxiety associated with vaccinations, facilitating their acceptance (208, 321).

Fifth, international cooperation and investment in vaccine development are expected to continue growing, especially in the face of the threat of emerging and re-emerging infectious diseases. Partnerships among governments, international organizations, the pharmaceutical industry, and academia will be essential for ensuring equitable vaccine access and expedited global distribution.

Sixth, enhancing health literacy and effective vaccine communication will be pivotal in increasing vaccine uptake and trust. While vaccine hesitancy is not a new phenomenon, it is a recurring challenge that has waxed and waned in parallel with advances in vaccinology. History has demonstrated that vaccines are one of the most powerful tools in humanity's arsenal against infectious diseases. Their continued success depends not only on scientific innovation but also on maintaining public trust and acceptance. As we move forward, it is imperative to learn from past experiences, both triumphs and setbacks, to ensure safe and effective vaccines are accessible for all.

## Author contributions

DM: Formal analysis, Investigation, Writing – original draft. RV: Formal analysis, Investigation, Writing – original draft. JV: Formal analysis, Investigation, Writing – original draft. LC: Formal analysis, Investigation, Writing – original draft. JT: Formal analysis, Investigation, Writing – review & editing. MB: Formal analysis, Investigation, Writing – original draft. Y-YT-R: Formal analysis, Investigation, Writing – original draft. AO: Formal analysis, Investigation, Writing – original draft. MO'R: Formal analysis, Writing – review & editing.

## In memoriam

This paper is dedicated to the memory of Carmenza Forero (1955–2020), beloved mother and greatest teacher.
